# A simplified model of NMDA-receptor-mediated dynamics in leaky integrate-and-fire neurons

**DOI:** 10.1007/s10827-025-00911-8

**Published:** 2025-08-05

**Authors:** Jan-Eirik Welle Skaar, Nicolai Haug, Hans Ekkehard Plesser

**Affiliations:** 1 Department of Data Science, Faculty of Science and Technology, Norwegian University of Life Sciences, Ås, Norway; 2 Department of Numerical Analysis and Scientific Computing, Simula Research Laboratory, Oslo, Norway; 3 Institute for Advanced Simulation (IAS-6), Jülich Research Centre, Jülich, Germany; 4 Käte Hamburger Kolleg: Cultures of Research (c:o/re), RWTH Aachen University, Aachen, Germany

## Abstract

A model for NMDA-receptor-mediated synaptic currents in leaky integrate-and-fire neurons, first proposed by Wang (J Neurosci, 1999), has been widely studied in computational neuroscience. The model features a fast rise in the NMDA conductance upon spikes in a pre-synaptic neuron followed by a slow decay. In a general implementation of this model which allows for arbitrary network connectivity and delay distributions, the summed NMDA current from all neurons in a pre-synaptic population cannot be simulated in aggregated form. Simulating each synapse separately is prohibitively slow for all but small networks, which has largely limited the use of the model to fully connected networks with identical delays, for which an efficient simulation scheme exists. We propose an approximation to the original model that can be efficiently simulated for arbitrary network connectivity and delay distributions. Our results demonstrate that the approximation incurs minimal error and preserves network dynamics. We further use the approximate model to explore binary decision making in sparsely coupled networks.

## Introduction

A model for a leaky integrate-and-fire neuron with NMDA-receptor-mediated synaptic currents generating persistent activity proposed by Wang and Brunel (Wang, 1999; Brunel & Wang, 2001; Wang, 2002), based on earlier kinetic modeling work by Destexhe et al. (1994), has been widely adopted in computational neuroscience, both for spiking-neuron and mean-field models (Wong & Wang, 2006; Deco & Jirsa, 2012). The model features a two-dimensional nonlinear system of ordinary differential equations for the presynaptic gating variable $S_j(t)$. Due to the nonlinearity of the system, the synaptic current in a postsynaptic neuron cannot be simulated in aggregated form. In a general implementation of the model, all synapses must be simulated explicitly, which is prohibitively expensive for all but small networks. In the specific case of a fully connected network with identical delays, the sum over all presynaptic gating variables is identical for all neurons and can be simulated globally instead of individually for each neuron. The original model is, therefore, mainly of use in the case of fully connected networks with identical delay.

Noting that the presynaptic gating variable $S_j(t)$ only depends on the spike history of the presynaptic neuron, we show that the NMDA dynamics can be approximated by an exponential decay between spikes and a history-dependent jump upon spikes. This form allows the summed gating variables in postsynaptic neurons to be reduced to a single variable, which can be efficiently simulated regardless of network connectivity or delay distribution. In the present work, we derive the approximate model and empirically characterize the error by comparing the original model and the approximation in neurons receiving identical input spikes. We show that errors in the synaptic currents vanish rapidly and that the effect on the membrane potential is small. Furthermore, we reproduce the binary decision-making network studied by Wang (2002) and Wong and Wang (2006) with both the approximate model and the original model. We find that the model dynamics are well-preserved in the approximation. Benchmarks show a significant speedup for the approximate model compared to a general implementation of the original model designed for arbitrary connectivity and delay distributions. Leveraging the flexibility and enhanced performance of the approximate model, we explore the dynamics of a sparsely connected binary decision-making network. A reference implementation of our approximate model is made available in the NEST simulator (Graber et al., 2024) as model iaf_bw_2001.

## Methods

In this section, we first describe the original model, followed by the derivation of the approximate model. We then present the network models used in this paper and our benchmarking setup.

### Description of the original model

The original model (Brunel & Wang, 2001) is a conductance-based leaky integrate-and-fire neuron with a synaptic NMDA current given by

1 $$\begin{aligned} \begin{array}{c} I_\textrm{NMDA}(t) = \\ \frac{ g_\textrm{NMDA} \times \left( V(t) - V_E \right) }{ 1 + \left[ \mathrm {Mg^{2+}} \right] \textrm{exp} \left( -0.062 V(t) \right) / 3.57} \\ \times \sum _{j=1}^{N_E} w_j S_{j,\textrm{NMDA}}(t) \end{array}\end{aligned}$$

2 $$\begin{aligned} \begin{array}{c} \frac{dS_{j,\textrm{NMDA}}(t)}{dt} = \\ -\frac{S_{j,\textrm{NMDA}}(t)}{\tau _\textrm{NMDA,decay}}+ \alpha x_j(t) \left( 1 - S_{j,\textrm{NMDA}}(t) \right) \end{array}\end{aligned}$$

3 $$\begin{aligned} \frac{dx_j(t)}{dt} = - \frac{x_j(t)}{\tau _\textrm{NMDA,rise}} + \sum _k \delta \left( t - t_j^k \right) \end{aligned}$$

where $\tau _\textrm{NMDA,decay}$, $\tau _\textrm{NMDA,rise}$ and $\alpha$ are model parameters, and $t_j^k$ are the spike times of neuron *j*. See Table 1 for the complete model equations and Table 2 for parameter values.

### Simplified NMDA gating dynamics

We will focus solely on the NMDA gating variables $S_j(t)$ and $x_j(t)$. For simplicity, we use the shorthand notation $\tau _\textrm{r}$ and $\tau _\textrm{d}$ to represent $\tau _\textrm{NMDA,rise}$ and $\tau _\textrm{NMDA,decay}$, respectively. Assuming that neuron *j* last spiked at time zero and does not spike again until time *t*, the solution to Eq. (3) is given by

4 $$\begin{aligned} x_j(t) = x_j^0 \textrm{exp}\left( -\frac{t}{\tau _\textrm{r}} \right) \textrm{,} \end{aligned}$$

where $x_j^0$ is the value immediately after the spike. By substituting the solution for $x_j$ into Eq. (2), we obtain the following expression for the time evolution of $S_j$ until *t*:

5 $$\begin{aligned} \frac{dS_{j}}{dt} + \left( \frac{1}{\tau _\textrm{d}} + \alpha x_j^0 \textrm{exp}\left[ -\frac{t}{\tau _\textrm{r}}\right] \right) S_{j}&= \alpha x_j^0 \textrm{exp}\left[ -\frac{t}{\tau _\textrm{r}}\right] \end{aligned}$$

We obtain the formal solution by applying an integrating factor as follows:

6 $$\begin{aligned} \begin{array}{c} S_{j}(t) = \textrm{exp}\left[ -\frac{t}{\tau _\textrm{d}} - \alpha x_j^{0} \tau _\textrm{r} \left( 1 -\textrm{exp}\left[ -\frac{t}{\tau _\textrm{r}} \right] \right) \right] \\ \times \left( S_{j}^0+ \alpha x_j^0 J(t) \right) \end{array}\end{aligned}$$

where $x_j^0$ and $S_j^0$ are the initial conditions. *J*(*t*) is the integral

$$\begin{aligned} J(t) = \int _0^{t} \textrm{exp}\left[ \frac{t'}{\tilde{\tau }} + \alpha x_j^0 \tau _\textrm{r} \left( 1 - \textrm{exp}\left[ -\frac{t'}{\tau _\textrm{r}} \right] \right) \right] dt' \end{aligned}$$

where $\tilde{\tau } = (1/\tau _\textrm{d} - 1 / \tau _\textrm{r})^{-1}$. This integral does not have a closed-form solution.

We seek an approximation of the form

7 $$\begin{aligned} \hat{S_j} (t) = S_\textrm{post} \textrm{exp}\left( -\frac{t}{\tau _d}\right) \end{aligned}$$

where $S_\textrm{post}$ is the—as yet unknown—initial value of the function immediately after spiking. We further assume that $x_j$ has decayed to 0 before neuron *j* fires its next spike, so that $x_j$ jumps to $x_j^0 = 1$ as the next spike is fired. We determine $S_\textrm{post}$ as a function of the value of $\hat{S_j} (t)$ immediately before spiking, by requiring that the approximation is asymptotically equal to the true solution, i.e.,

8 $$\begin{aligned} \lim _{t \rightarrow \infty }\frac{S_j(t)}{\hat{S_j} (t)} = 1 \end{aligned}$$

By substituting Eqs. (6) and (7) into Eq. (8), we find that

9 $$\begin{aligned} \begin{array}{c} S_\textrm{post} = \lim _{t \rightarrow \infty } \textrm{exp}\left[ - \alpha \tau _\textrm{r} \left( 1-\textrm{exp}\left[ -\frac{t}{\tau _\textrm{r}} \right] \right) \right] \\ \times \left( S_{j}^0 + \alpha \tilde{J}(t) \right) \end{array}\end{aligned}$$

where

10 $$\begin{aligned} \tilde{J}(t) = \int _0^{t} \textrm{exp}\left[ \frac{t'}{\tilde{\tau }} + \alpha \tau _\textrm{r} \left( 1 - \textrm{exp}\left[ -\frac{t'}{\tau _\textrm{r}} \right] \right) \right] dt' \end{aligned}$$

Substituting $u = \alpha \tau _r e^{-\frac{t'}{\tau _r}}$, the integral $\tilde{J}(t)$ can be expressed in the limit as

$$\begin{aligned} \lim _{t \rightarrow \infty } \tilde{J}(t)&= \frac{1}{\alpha }e^{\alpha \tau _r}(\alpha \tau _r)^{\frac{\tau _r}{\tau _d}} \int _0^{\alpha \tau _r} u^{-\frac{\tau _r}{\tau _d}} e^{-u}du \\&= \frac{1}{\alpha }e^{\alpha \tau _r}(\alpha \tau _r)^{\frac{\tau _r}{\tau _d}} \gamma \big [1 - \frac{\tau _r}{\tau _d}, \alpha \tau _r \big ] \textrm{,} \end{aligned}$$

where $\gamma$ is the lower incomplete gamma function (DLMF, 2024, Eq. 8.2.1). Thus, Eq. (9) can be evaluated as

11 $$\begin{aligned} S_\textrm{post} = e^{-\alpha \tau _\textrm{r}} S_j^0 + (\alpha \tau _r)^{\frac{\tau _r}{\tau _d}} \gamma \big [1 - \frac{\tau _r}{\tau _d}, \alpha \tau _r \big ] \end{aligned}$$

We define two constants

12 $$\begin{aligned} k_0&= \left( \alpha \tau _r \right) ^{\frac{\tau _r}{\tau _d}} \gamma \left[ 1 - \frac{\tau _r}{\tau _d}, \alpha \tau _r \right] \end{aligned}$$

13 $$\begin{aligned} k'_1&= e^{-\alpha \tau _\textrm{r}} \;, \end{aligned}$$

which depend solely on the synaptic parameters. For a presynaptic neuron *j*, let $\hat{t}$ be the time of the previous spike and $t^-$ the time immediately before the next spike. Then, according to the definition of our approximation, we have

$$\begin{aligned} S_j \left( t^- \right) = S_j \left( \hat{t} \right) e^{-\frac{t^- - \hat{t}}{\tau _d}} \;. \end{aligned}$$

The value of $S_j$ at $t^+$, immediately after the spike, is then given by

$$\begin{aligned} S_j \left( t^+ \right) = S_\textrm{post} = k_0 + k'_1 S_j \left( t^- \right) \;. \end{aligned}$$

In a postsynaptic neuron, the sum over all presynaptic $S_j$ is aggregated in a single variable. Therefore, the change in $S_j$ upon the spike, rather than its value immediately after the spike, must be transmitted to the postsynaptic neuron to update the aggregated variable. The change in $S_j$ upon the spike at time *t* is

$$\begin{aligned} \Delta S_j&= S_j \left( t^+ \right) - S_j \left( t^- \right) \\&= k_0 + k'_1 S_j \left( t^- \right) - S_j \left( t^- \right) \\&= k_0 + k_1 S_j \left( t^- \right) \textrm{,} \end{aligned}$$

with $k_1 = k'_1 - 1$. The change $\Delta S_j$ can then be transmitted to all postsynaptic neurons and added to their aggregated *S* input variable. The aggregated NMDA gating variable in a postsynaptic neuron can be simulated using the following differential equation

14 $$\begin{aligned} \frac{dS}{dt} = -\frac{S}{\tau _\textrm{d}} + \sum _{j,k} \Delta S_j(t) \delta \left( t - t_j^k \right) \;. \end{aligned}$$

Reference implementations of both the approximate model and the original model can be found in the NEST simulator under the model names iaf_bw_2001 and iaf_bw_2001_exact, respectively.

### Network models

Here we describe the network model used to validate the approximation and for exploring decision-making in sparsely connected networks respectively, as well as our benchmarking setup.

#### Decision-making network

**Table 1:** Description of decision-making network following the guidelines of Nordlie et al. (2009)

A	Model summary				
Populations	Three excitatory, one inhibitory, three external				
Network model	Fully connected				
Neuron model	Local populations: leaky integrate-and-fire, external: Poisson generator				
Synapse model	Conductance-based, with fixed strength for each pair of populations				
B	Populations				
Name	Symbol	Size			
Selective A	$E_\textrm{A}$	$N_\textrm{A} = f N_\textrm{E}$			
Selective B	$E_\textrm{B}$	$N_\textrm{B} = f N_\textrm{E}$			
Nonselective	$E_\textrm{N}$	$N_\textrm{N} = (1 - 2f) N_\textrm{E}$			
Inhibitory	*I*	$N_\textrm{I}$			
C	Connectivity				
Source	Target	Weight	Delay	Receptors	Connection rule
$E_\textrm{A}$	$E_\textrm{A}$	$w_+$	$t_\textrm{d}$	AMPA,NMDA	Fully connected
$E_\textrm{B}$	$E_\textrm{B}$	$w_+$	$t_\textrm{d}$	AMPA,NMDA	Fully connected
$E_\textrm{A}, E_\textrm{N}$	$E_\textrm{B}$	$w_-$	$t_\textrm{d}$	AMPA,NMDA	Fully connected
$E_\textrm{B}, E_\textrm{N}$	$E_\textrm{A}$	$w_-$	$t_\textrm{d}$	AMPA,NMDA	Fully connected
$E_\textrm{A}, E_\textrm{B}, E_\textrm{N}$	$E_\textrm{N}, I$	1	$t_\textrm{d}$	AMPA,NMDA	Fully connected
*I*	$E_\textrm{A}, E_\textrm{B}, E_\textrm{N}, I$	1	$t_\textrm{d}$	GABA	Fully connected
D	Neuron model				
Type	Leaky integrate-and-fire neuron				
Description	Dynamics of membrane potential $V_i(t)$ (neuron $i\in [1,N]$):				
	- Spike emission at times $t^i_l$ with $V_i(t^i_l)\ge V_{\text {thr}}$				
	- Subthreshold dynamics:				
	$$C_\textrm{m}\frac{\textrm{d}V_i}{\textrm{d}t} = -g_\textrm{m}(V_i - E_\textrm{L}) - I_i(t) \quad \forall l:\,t\notin (t^i_l,t^i_l+t_\text {ref}] \qquad \qquad \qquad \qquad \text {(15)}$$				
	where $C_\textrm{m}$ is the membrane capacitance, $V_i$ the membrane potential, $g_\textrm{m}$ the membrane conductance, and $I_i(t)$ the synaptic inputs.				
	- Reset + refractoriness: $V_i(t)= V_\text {reset}$ $\forall l:\,t\in (t^i_l,t^i_l+t_\text {ref}]$				
	Solved with RKF45 with adaptive step size, where spikes are checked at intervals of *dt*.				
	Membrane potential is initialized as $V_i = E_\textrm{L}$ at $t=0$.				
E	Synapse model				
Type	Conductance-based currents				
Description	$I_\textrm{syn}(t) = I_\textrm{AMPA}(t) + I_\textrm{NMDA}(t) + I_\textrm{GABA}(t) \textrm{,}$				
	$$I_\textrm{AMPA}(t) = g_\textrm{AMPA}\times (V(t) - V_E) \times \sum \limits _{j=1}^{N_E}w_jS_{j,\textrm{AMPA}}(t) \textrm{,}$$				
	$$I_\textrm{NMDA}(t) = \frac{g_\textrm{NMDA}\times \left( V(t) - V_E \right) }{1+[\mathrm {Mg^{2+}}]\textrm{exp}\left( -0.062V(t) \right) /3.57}\times \sum \limits _{j=1}^{N_E}w_jS_{j,\textrm{NMDA}}(t) \textrm{,}$$				
	$$I_\textrm{GABA}(t) = g_\textrm{GABA}\times \left( V(t) - V_I \right) \times \sum \limits _{j=1}^{N_E}w_jS_{j,\textrm{GABA}}(t)$$				
	$$\frac{dS_{j,\textrm{AMPA}}}{dt} = -\frac{S_{j,\textrm{AMPA}}}{\tau _\textrm{AMPA}}+\sum \limits _k \delta \left( t - t_j^k \right)$$				
	$$\frac{dS_{j,\textrm{GABA}}}{dt} = -\frac{S_{j,\textrm{GABA}}}{\tau _\textrm{GABA}} + \sum \limits _k \delta \left( t - t_j^k \right)$$				
	$$\left. \begin{array}{c}\frac{dS_{j,\textrm{NMDA}}}{dt} = -\frac{S_{j,\textrm{NMDA}}}{\tau _\textrm{NMDA,decay}}+ \alpha x_j \left( 1 - S_{j,\textrm{NMDA}} \right) \\ \frac{dx_j}{dt} = - \frac{x_j}{\tau _\textrm{NMDA,rise}} + \sum \limits _k \delta \left( t - t_j^k \right) \end{array}\right\} \quad \textrm{Exact model}$$				
	$$\left. \frac{dS_{j,\textrm{NMDA}}}{dt} = -\frac{S_{j,\textrm{NMDA}}}{\tau _\textrm{NMDA,decay}} + \sum \limits _k \delta \left( t - t_j^k \right) \left( k_0 + k_1 S_{j,\textrm{NMDA}}\right) \right\} \quad \textrm{Approximation}$$				
F	Signals				
Name	Target	Description			
$\mathrm {P_0}$	$E_\textrm{A}, E_\textrm{B}, E_\textrm{N}, I$	Constant rate Poisson generator with rate $\nu _\textrm{ext}$ and weight 1. Active from $t=0$ to $t=T$.			
$\mathrm {P_A}$	$E_\textrm{A}$	Poisson generator with rates sampled from $\mathcal {N}(\mu _1, 4)$ every $50 \textrm{ms}$ and weight 1. Active from $t=1000\ \textrm{ms}$ to $t=3000\ \textrm{ms}$.			
$\mathrm {P_B}$	$E_\textrm{B}$	Poisson generator with rates sampled from $\mathcal {N}(\mu _2, 4)$ every $50 \textrm{ms}$ and weight 1. Active from $t=1000\ \textrm{ms}$ to $t=3000\ \textrm{ms}$.

**Table 2:** Parameters for decision-making network from Wang (2002)

Symbol	Description		
Neuron parameters			
		Excitatory neurons	Inhibitory neurons
$C_\text {m}$	Membrane capacitance	500 pF	250 pF
$t_\text {ref}$	Absolute refractory period	2 ms	1 ms
$V_{\text {thr}}$	Firing threshold	$-50$ mV	$-50$ mV
$V_\text {reset}$	Reset membrane potential	$-55$ mV	$-55$ mV
$E_L$	Passive leak reversal potential	$-70$ mV	$-70$ mV
$\alpha$	NMDA gating variable gain factor	0.5 ms^-1^	0.5 ms^-1^
$[\textrm{Mg}^{2+}]$	Magnesium ion concentration	1.0 mM	1.0 mM
$\tau _\textrm{AMPA}$	AMPA synaptic time constant	2 ms	2 ms
$\tau _\textrm{GABA}$	GABA synaptic time constant	2 ms	2 ms
$\tau _\textrm{NMDA,r}$	NMDA synaptic rise time constant	2 ms	2 ms
$\tau _\textrm{NMDA,d}$	NMDA synaptic decay time constant	100 ms	100 ms
$g_\textrm{L}$	Leak conductance	25 nS	20 nS
$g_\textrm{AMPA}$	AMPA conductance	0.05 nS	0.04 nS
$g_\textrm{GABA}$	GABA conductance	1.3 nS	1.0 nS
$g_\textrm{NMDA}$	NMDA conductance	0.165 nS	0.13 nS
Population parameters			
$N_\textrm{E}$	Total number of excitatory neurons	1600	
$N_I$	Total number of inhibitory neurons	400	
*f*	Fraction of each selective population	0.15	
Connection parameters			
$t_\text {d}$	Synaptic delay period	0.5 ms	
$w_+$	Potentiated weight	1.7	
$w_-$	Depressed weight	$1-f(w_+ - 1)(1 - f)$	
Signal parameters			
$\nu _\textrm{ext}$	External input rate	2400 sp/s	
$\mu _0$	Base signal rate to selective populations	40 sp/s	
$\rho _\textrm{A}$	Coherence scaling factor selective pop. A	0.4	
$\rho _\textrm{B}$	Coherence scaling factor selective pop. B	0.4	
Simulation parameters			
$T_\textrm{sim}$	Simulation duration	4000 ms	
*dt*	Time resolution	0.1 ms	

To validate the approximation in a practical use case, we replicate the decision-making network model originally studied by Wang (2002). This network consists of three excitatory populations and one inhibitory population, all of which are recurrently connected. An external population, modeled as a Poisson process, projects equally onto all recurrent neurons. The selective populations $E_\textrm{A}$ and $E_\textrm{B}$ each comprise a fraction *f* of the total number of excitatory neurons, while the nonselective population $E_\textrm{N}$ comprises the remaining fraction $1 - 2f$ of excitatory neurons. The selective populations receive a transient stimulus in the form of spikes onto AMPA synapses from an additional Poisson process. The relative strength of the transient stimulus received by the selective populations is determined by the input coherence $c'$ of the signal. The rate of the transient stimulus is given by $\mu _\textrm{A} = \mu _0 + \rho _\textrm{A} c'$, $\mu _\textrm{B} = \mu _0 - \rho _\textrm{B} c'$. In this study, we consider the case where $\mu _0 = 40$ sp/s, and $\rho _\textrm{A} = \rho _\textrm{B} = \frac{\mu _0}{100}$. 

A concise description of the model is provided in Table 1, and the parameter values used are listed in Table 2.

#### Sparse decision-making network

To investigate the dynamics of a sparsely connected decision-making network, we change the connectivity rule to random fixed in-degree $\epsilon _\textrm{XY} N_\textrm{X}$ without multapses (Senk et al., 2022). Here, $\epsilon _\textrm{XY}$ denotes the connection probability from presynaptic population *X* onto postsynaptic population *Y*, and $N_X$ denotes the size of presynaptic population *X*. When $\epsilon _\textrm{XY} = 1$ for all $\textrm{X}$ and $\textrm{Y}$, the fully connected network is recovered.

For the first two seconds of the simulations, all outgoing NMDA connections from the selective populations are replaced by a constant “$S_\textrm{NMDA}$-current” with value $N_\textrm{A} \epsilon _\textrm{AX}w_\textrm{AX}g_\textrm{NMDA} / \tau _\textrm{NMDA}$, which is added to the right-hand side of Eq. 14 for all populations. Here, $\epsilon _\textrm{AX}$ is the connection probability from presynaptic selective population A onto postsynaptic population X, and $w_\textrm{AX}$ is the corresponding synaptic weight. This drives the aggregated $S_\textrm{NMDA}$-value in the postsynaptic neurons towards the value it would take if all $S_{j,\textrm{NMDA}}$-values in selective population A were 1, and the corresponding values for selective population B were 0, effectively clamping them. After 2 seconds, the NMDA connections are restored and the “$S_\textrm{NMDA}$-current” is removed. If the given connectivity admits an asymmetric state, i.e., a state where one of the selective populations has higher activity than the other, the network will relax into it.

By varying the values of the connection probabilities within the network, we can determine the values that support decision-making dynamics. Simulations were run with $\epsilon _\textrm{XY} = 0.2$ for all pairs of populations except $\epsilon _\textrm{AA} = \epsilon _\textrm{BB}$ (internal connectivity within each selective population) and $\epsilon _\textrm{IA} = \epsilon _\textrm{IB}$ (inhibitory-selective connectivity). These two connectivity values were systematically varied across 33 evenly spaced points in the interval [0.2, 1.0] for each pair.

#### Benchmarking setup

We use the fully-connected decision-making network to measure simulation times, but with $f=0$. This results in a network with one excitatory and one inhibitory population and steady-state activity. We scale the network size from 1.28 times to 10.24 times the size of the original network in powers of 2. These network sizes, comprising from 2560 to 20480 neurons, were chosen to be evenly divisible across up to 128 parallel threads. Due to the all-to-all connectivity and the pairing of AMPA and NMDA synapses, this results in a network of about 755 million synapses for the largest network size. Synaptic conductances for all recurrent connections were scaled inversely with network size to approximately maintain network dynamics.

We benchmark four different implementations of this model: Using NEST and our approximation (iaf_bw_2001), using NEST and the original model (iaf_bw_2001_exact) as well as two implementations in Brian2 as external references. The first of these is a restricted implementation that only supports fully connected networks with equal delays (Wimmer & Stimberg, 2023), while the other allows arbitrary connectivity; it is inspired by Moreni et al. (2025). Neuron models in NEST used an adaptive Runge-Kutta-Fehlberg-45 numerical solver, which has a slightly higher computational cost than the Runge-Kutta-4 numerical solver used in the Brian2 simulations.

Benchmark results reported here were obtained with NEST 3.8 (Graber et al., 2024) on the JURECA supercomputer at the Jülich Supercomputing Center equipped with AMD EPYC 7742 CPUs providing 128 compute cores. Simulations were performed using 8 and 128 threads. Eight threads are the minimum required to simulate the largest network size with NEST.

## Results

We assess the accuracy of the approximate model using two approaches. First, we examine the differences in NMDA current and membrane potential between neurons with exact and approximate NMDA dynamics when they receive synaptic activations from a Poisson process at different rates. Second, we reproduce the binary decision-making network studied by Wang (2002) and Wong and Wang (2006) using both the exact and approximate models to compare the dynamics of each network. Furthermore, leveraging the flexibility and improved performance offered by the approximation, we explore the dynamics of the binary decision-making network with sparse connectivity.

### Errors in NMDA-receptor-mediated currents and membrane potential

**Fig. 1:**
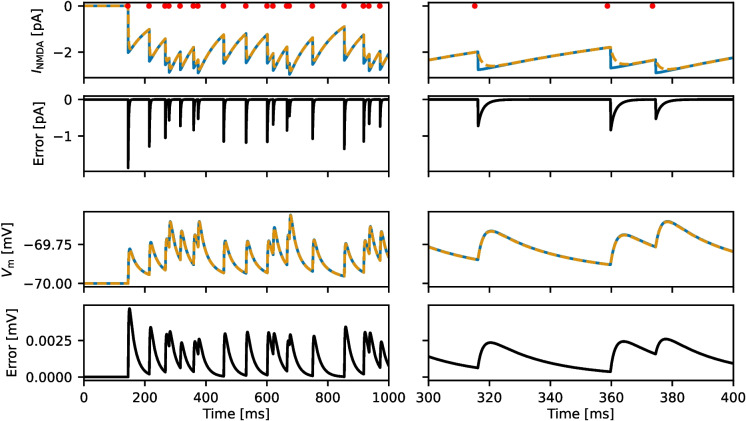
NMDA-receptor-mediated synaptic currents and membrane potential. For both the exact model (dashed orange lines) and the approximate model (solid blue lines), a postsynaptic neuron receives identical input spikes. The resulting NMDA receptor-mediated synaptic currents and the error $I_\textrm{NMDA,exact} -I_\textrm{NMDA,approximate}$ is shown in the top half, while the membrane potential and corresponding error is shown in the bottom half. The red dots indicate the spike arrival times. The plots on the right side shows the same data as on the left, but zoomed in to provide a clearer view of the effect of individual spikes

While the original model has a finite rise time in the NMDA gating variable, characterized by the time constant $\tau _\textrm{NMDA,r}$, the approximate model introduces an instantaneous jump. Immediately after any given spike, the error in the gating variable of the approximation will then be $k_0 + k_1 S_0$, where $S_0$ is the value of the gating variable immediately before the spike. In a postsynaptic neuron, the effect on the synaptic current due to NMDA receptors will be through the coupling described by Eq. (1), and the error of the synaptic current will also rise instantaneously. The errors of the membrane potential are filtered through Eq. (15) and will increase at a finite rate. Due to the instantaneous jump in the NMDA gating variable caused by a spike, the postsynaptic current will be higher in the approximate model compared to the exact model. As the gating variable of the exact model increases, the error rapidly decreases on the time scale of $\tau _\textrm{NMDA,r}$. Figure 1 shows an example of $I_\textrm{NMDA}$ and $V_\textrm{m}$ for both the exact and approximate models in a simulation where they receive identical input at 20 sp/s. For the synaptic currents, the error jumps instantaneously and decays on a short time scale. The error in membrane potential rises on a short time scale and decays over a longer time scale.

Because the error of the approximate NMDA gating variable from a single spike decreases rapidly and its differential equation is linear, the errors do not accumulate over time but instead depend only on the number of spikes in the immediate past. If the neuron receives sufficiently high input to reach the threshold, the small differences between the exact model and the approximate model will result in small changes to the exact timing of spike events. These errors in spike times accumulate, and in such cases, the differences between simulations of the exact model and the approximate model receiving identical input become more pronounced.

**Fig. 2:**
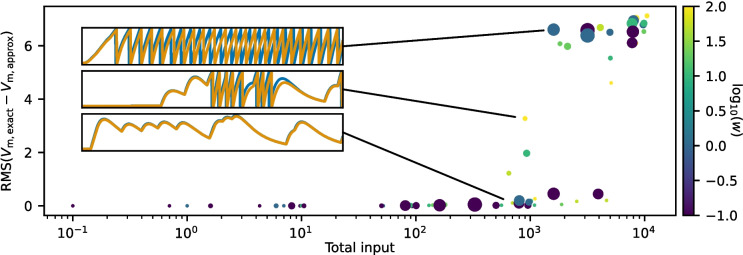
RMS of difference in voltage traces. The root mean square (RMS) of the difference between the voltage traces of the exact and approximate models in a simulation where a postsynaptic neuron receives NMDA currents from a presynaptic population of Poisson neurons. The color of the dots indicates the synaptic connection weight (*w*), ranging from 0.1 to 100. The size of each dot is proportional to the square root of the presynaptic population size $n_\textrm{pre}$, varying from 1 to 3200. The total input is calculated as $n_\textrm{pre} \nu _\textrm{pre} w$, where $\nu _\textrm{pre}$ is the presynaptic firing rate. The three insets show the time series of the membrane potential of the exact model (orange) and the approximation (blue) in simulations where the neuron respectively exhibits high, low, and no spiking activity

Figure 2 shows the root mean square difference (RMS) between membrane potentials from simulations of the exact model and the approximate model. Both models receive identical inputs from a presynaptic population with Poisson spikes, with variations in presynaptic population size, presynaptic firing rate, and synaptic connection weights. As the total input—determined by the product of the number of presynaptic neurons, their firing rate, and the synaptic connection weight—increases, the RMS difference remains minimal until the postsynaptic neuron begins to fire. Once the postsynaptic neuron fires, the difference in membrane potential between the exact model and the approximate model increases rapidly due to the differences in spike times. The NMDA gating variable in the approximate model is higher than in the exact model, causing the approximate model to spike slightly earlier when given identical input.

### Reproducing the Wang (2002) binary decision-making network

In the binary decision-making network studied by Wang (2002) and Wong and Wang (2006), described in Section 2.3.1, NMDA receptor-mediated synaptic currents are crucial for enabling the network to sustain high activity within the selective populations. During the period when the selective populations receive a transient stimulus, the network transitions into an asymmetric state, characterized by high activity in one of the selective populations and low activity in the other. After the transient stimulus period, the network may either maintain the asymmetric state or, with a certain probability, revert to a state where both populations have equal activity.

**Fig. 3:**
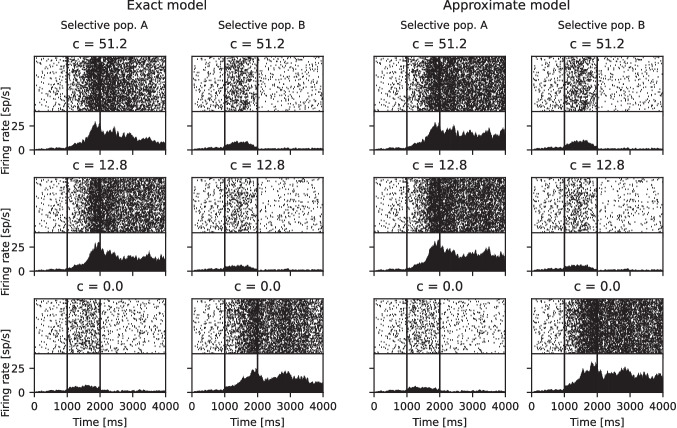
Comparison of the exact and approximate models in binary decision-making network dynamics. Simulations of a binary decision-making network using both the exact and the approximate models for three different coherence levels. The network connectivity, external inputs, and stimuli are identical across both models. Each panel shows a spike raster of 100 neurons from selective populations A and B at the top, and the population activity, measured by averaging the summed population spiking histogram over 50 ms time bins, at the bottom. The vertical lines indicate the start and end of the transient stimulus

Figure 3 compares the dynamics of the binary decision-making network when modeled using the exact and approximate models. The figure shows three example simulations of the network at different coherence levels, similar to Figure 2 in Wang (2002). The network dynamics are qualitatively similar for both the exact and approximate models; however, for the approximate model, the selective population with higher activity shows slightly increased activity. This is due to the marginally higher values of the NMDA gating variable in the approximate model. At coherence level $c' = 0$, both selective populations have an equal probability of transitioning into the high activity state. As coherence increases, the stimulus to selective population A also increases, thereby increasing the probability that the network will transition into a state where population A has the higher activity. By performing multiple simulations at different coherence levels, the probability of making the correct choice—which is defined as selecting population A for $c'> 0$ because it receives a stronger stimulus—can be determined as a function of the coherence level.

**Fig. 4:**
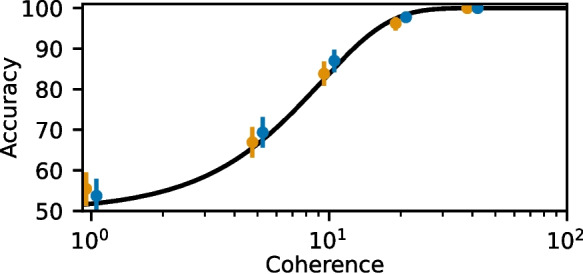
Decision-making accuracy. The probability of the network making the correct decision—defined as selective population A maintaining higher activity after the stimulus is turned off—as a function of the coherence level, $c'$. For each coherence level $c' \in \{1, 5, 10, 20, 40\}$, the probability is calculated by performing 400 simulations for both the exact (orange dots) and the approximate (blue dots) models and determining the proportion of simulations where population A wins. The data points are slightly offset along the x-axis for clarity, although they are all simulated with the same coherence level value. A 90% confidence interval, estimated by bootstrapping, is represented by the error bars. The black line shows the Weibull function fitted by Wang (2002) for the same experimental setup

Figure 4 shows the probability of making a correct choice at different levels of coherence after 2 seconds of stimulation as in Figure 4 of (Wang, 2002). For both the exact and approximate models, 400 simulations were run at coherence levels $c' \in \{1, 5, 10, 20, 40\}$. The winner is determined as the selective population with the highest activity after the stimulus period. A 90% confidence interval is estimated using bootstrapping by independently resampling the simulations 5000 times with replacement and calculating the probability for each trial. The error bars represent the confidence interval at each coherence level. Our results for the exact and approximated model agree well with the results from Wang (2002), which are shown by the black line in Fig. 4. It shows the Weibull function $P(\textrm{correct}) = 1 - 0.5 \times \textrm{exp}(-(\frac{c'}{\alpha })^\beta )$ for parameters $\alpha = 9.2$ and $\beta = 1.5$ reported by Wang (2002) as optimal fit to their simulation results.

### Benchmarks

**Fig. 5:**
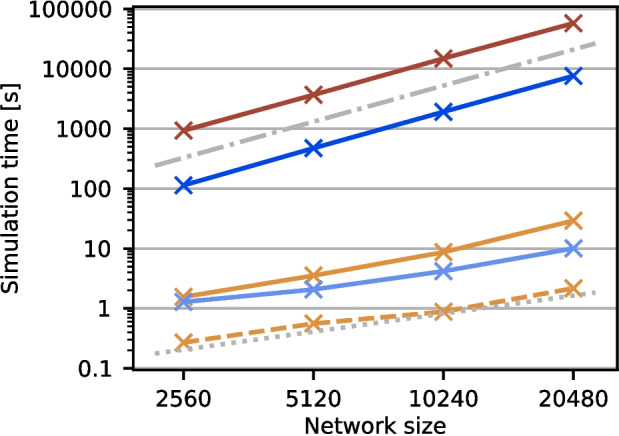
Network simulation time for different model implementations and network sizes. Solid lines show the time required to simulate 1 s model time for the exact implementation (dark brown) and approximation (light brown) in NEST, and the unrestricted (dark blue) and restricted (light blue) implementations in Brian2, all simulated on eight threads. The dashed light brown line shows the NEST approximation for 8 MPI processes with 16 threads each. The dash-dotted grey line marks a quadratic, the dotted grey line a linear increase in simulation time with network size. Data are from a single simulation per data point. As the data are very clear, evolve systematically with size and the different implementations differ by factors, we prioritized energy conservation rather than collecting statistics.

**Table 3:** Simulation times for the largest network size

	NEST		Brian2	
Exact	Approximation	Approx ($8\times 16$)	Unrestricted	Restricted
57659.4 s	29.2 s	2.2 s	7610.1 s	10.1 s

Data are shown for all four implementation for eight threads and for the approximation in NEST also for 8 MPI processes with 16 threads each. These are the same data as the rightmost data points in Fig. 5

Figure 5 summarizes our benchmark experiments, with detailed timings for the largest network size provided in Table 3. The implementations in NEST (dark brown) and Brian2 (dark blue) supporting arbitrary connectivity and using the exact NMDA neuron model are more than two orders of magnitude slower than the approximating (NEST, light brown) and restricted (Brian2, light blue) implementations, reducing the wall-clock time required for simulating one second of model time from hours to seconds. Simulation times scale quadratically with network size for the general exact case in both NEST and Brian2, because the effort to integrate NMDA dynamics increases quadratically. Brian2 is faster than NEST in this case (7.6 times for the largest network size), presumably because Brian2 generates more efficient code by just-in-time compilation compared to NEST’s prebuilt binary. The choice of ODE integrator has a minor effect (data not shown) as has the slight difference in firing rates we observed^1^.

Using the same amount of computational resources (eight threads or CPU cores), the approximation in NEST is about 2.9 times slower than the restricted implementation in Brian2. Exploiting NEST’s hybrid parallelization capabilities (Plesser et al., 2007), we found 8 MPI processes using 16 threads each to be the optimal configuration to use all 128 cores available on a compute node. With this configuration, NEST simulated the largest network size in 2.2 s for 1 s of model time, i.e., about 4.6 times faster than the restricted and 3460 times faster than the unrestricted model in Brian2. Brian2 does not support MPI parallelization and we did not observe any improvement in simulation times beyond eight threads in a single process (data not shown).

Interestingly, the MPI-parallel simulation of the approximation in NEST shows linear scaling of simulation time with problem size, even though the number of synapses in the network grows quadratically also in this case. The most plausible explanation for this observation is that integrating the dynamics of the neurons including their aggregated synaptic conductances dominates computation in this case, while the actual cost of spike transmission becomes negligible.

### Binary decision-making in sparsely coupled networks

**Fig. 6:**
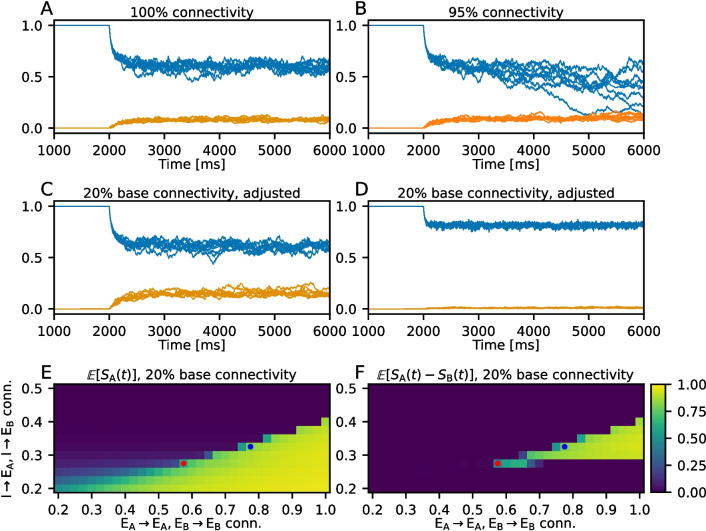
Decision-making dynamics in a sparsely coupled network. **(A)** Population-averaged values of $S_\textrm{A}(t)$ (blue lines) and $S_\textrm{B}(t)$ (orange lines) from eight differently seeded simulations of a fully connected network. **(B)** Same as (A), but for a network with 95% connectivity, where the recurrent conductances are scaled by a factor of 1/0.95. **(C)** and **(D)** Same as (A) and (B), but for a network with 20% base connectivity, where the intra-selective connectivity and inhibitory-selective connectivity have been adjusted; see text for details. The values of these connectivities are indicated by the red and blue dots respectively in (E) and (F). **(E)** Expectation of the $S_\textrm{A}(t)$ for times $\ge 4\,000~\textrm{ms}$, for a base connectivity of 20%, where the intra-selective and inhibitory-selective connectivities are adjusted. The values are also averaged over eight independent simulations. **(F)** Similar to (E), but for the difference $S_\textrm{A}(t) - S_\textrm{B}(t)$

Since our approximation allows for arbitrary network connectivity, we investigate the dynamics of a decision-making network with sparse connectivity, described in Section 2.3.2. To determine whether a particular connectivity supports decision-making, we clamp the value of $S_{j,\textrm{NMDA}}$ of all outgoing NMDA-synapses from selective population A to 1, and from selective population B to 0 for two seconds. We denote the population-averaged value of $S_{j,\textrm{NMDA}}$ over all neurons in selective population A by $S_\textrm{A}(t)$, and equivalently for selective population B. Once the clamp is released, the network dynamics are allowed to evolve freely, and $S_\textrm{A}(t)$ decreases while $S_\textrm{B}(t)$ increases. If a stable, asymmetric state where $\mathbb {E}[S_\textrm{A}(t)]> \mathbb {E}[S_\textrm{B}(t)]$ exists, the network will relax into this state.

Figure 6A and B show $S_\textrm{A}(t)$ and $S_\textrm{B}(t)$ from the decision-making network with 100% and 95% connectivity between all populations, respectively. Eight simulations with different seeds were run, and the individual time series are shown in the figures. In the 95% connectivity simulations, synaptic weights were scaled by a factor of 1/0.95. Even with this minor reduction in connectivity, the asymmetric state becomes unstable, leading the network to converge toward a symmetric state. Without scaling the weights, a mere one percent decrease in connectivity suffices to destabilize the asymmetric state.

The stability of the asymmetric state depends on the winning selective population’s ability to maintain a high NMDA current while ensuring sufficient recurrent inhibition to suppress the losing population. To explore this, we performed simulations of a sparsely connected network where the internal connectivity within each selective population, as well as the connectivity between inhibitory and selective populations, were systematically varied. Starting with a base connectivity of $20\%$, simulations were run with adjustments to intra-selective ($E_\textrm{A}\rightarrow E_\textrm{A}$ and $E_\textrm{B} \rightarrow E_\textrm{B}$, see Table 1C) and inhibitory-selective connectivity ($I \rightarrow E_\textrm{A}$ and $I \rightarrow E_\textrm{B}$), taking independent values on the interval [0.2, 1.0]. Figures 6C and 6D show examples from simulations with 20% base connectivity, but where the intra-selective and inhibitory-selective connectivities have been adjusted to regain decision-making dynamics. The values of the adjusted connectivities are indicated by the red and blue dots in Fig. 6E and F respectively. For the example in Fig. 6C, the network is in a state similar to that of the fully connected network shown in 6A. In contrast, the example in Fig. 6D, which features higher intra-selective connectivity, demonstrates a significantly larger difference between $S_\textrm{A}$ and $S_\textrm{B}$.

Figure 6E and F show the expected value of $S_\textrm{A}(t)$ and the expected value of $S_\textrm{A}(t) - S_\textrm{B}(t)$, respectively, taken over the time interval $[4000~\textrm{ms}, 6000~\textrm{ms}]$, and averaged over the eight independent simulations. Figure 6E illustrates that the expected value of $S_\textrm{A}$ is highly contingent upon both inhibitory-selective connectivity and intra-selective connectivity. As inhibitory connectivity increases, $S_\textrm{A}(t)$ quickly becomes almost entirely suppressed above a certain threshold, which is roughly linearly dependent on the selective connectivity. The expected difference between $S_\textrm{A}(t)$ and $S_\textrm{B}(t)$, shown in Fig. 6F, reveals the required balance between intra-selective and inhibitory-selective connectivity for the network to support decision-making dynamics. A minimum level of both inhibitory and selective connectivity is required, and, generally, for any given level of inhibitory connectivity, increasing the selective connectivity increases the distance $S_\textrm{A} - S_\textrm{B}$. For low levels of inhibitory-selective connectivity, $\mathbb {E}[S_\textrm{A}] = \mathbb {E}[S_\textrm{B}]$, with values increasing with the intra-selective connectivity. There are sharp upper and lower boundaries given by the level of inhibitory-selective connectivity, between which the two selective populations take on different levels of activity. Below the lower boundary, both populations are in a high activity state, and above the upper boundary, both populations are suppressed. It is important to note that synaptic conductances were kept constant in these simulations; varying them would likely change the connectivity balance needed to maintain decision-making dynamics.

## Discussion

In the present work, we have developed an approximate model of the NMDA-receptor-mediated synaptic currents proposed by Wang (1999), Brunel and Wang (2001), and Wang (2002) for leaky integrate-and-fire neurons. The original model features a gating variable that is modeled by a two-dimensional system of ordinary differential equations, for which the total synaptic inputs from a presynaptic population cannot be simulated in aggregated form, except for in the case of fully connected networks with identical delays. Two features of the original model are important to capture. First, the long time constant of the gating variable which is an order of magnitude larger than those of AMPA or GABA synapses, and second, the saturation of the gating variable as its value increases.

The approximate model is constructed such that it follows a simple exponential function between synaptic events, and with instantaneous rise times at all synaptic events while being asymptotically equal to the original model. This allows the sum over postsynaptic gating variables to be reduced to a single differential equation per neuron. The decay time constant is set explicitly to be the same as that of the original value. Since the rise time of the gating variable is typically taken to be very short compared to the decay time, reducing it to an instantaneous jump is not a large deviation from the original model. The magnitude of the jump in the gating variable after a synaptic event decreases as the value of the gating variable itself increases, such that it saturates, although the maximum value the approximate value can reach is slightly larger than one. As such, the interpretation of the gating variable as the fraction of open ion channels is not technically valid, but for practical purposes it is not important.

The error introduced by the approximation is mainly limited to the first few milliseconds after synaptic events and rapidly decays after (Fig. 1). Due to the instantaneous jump, the approximation gives higher values than the original model, which causes the NMDA currents to also be higher. Reducing the conductance slightly can help correct this, although in practice the effect is negligible. In the membrane potential, the subthreshold errors introduced by the approximation are less noticeable, as they are exponentially filtered with the larger membrane time constant. For the case of both models receiving identical input strong enough to cause spiking, there will be an error in the membrane potential that accumulates as the spike times are shifted (Fig. 2). In networks, this effect frequently will not be relevant, as sources of stochasticity such as Poissonian input and randomized connections will have a much stronger effect on exact spike timings. For the case of the binary decision-making network studied by Wang (2002) and Wong and Wang (2006), we show that the dynamics of the network are preserved (Fig. 3), and closely replicate the overall decision-making results (Fig. 4).

The approximate model reduces simulation time for network models with arbitrary connectivity by over two orders of magnitude, from hours to seconds per second of model time. Fully exploiting NEST’s parallel capabilities, we achieved simulation times only slightly longer than twice the time simulated. This significant improvement enables the systematic exploration of larger networks with plausible network structure, overall size and connection density including the NMDA dynamics introduced by Wang (2002). In contrast, the restricted implementation can only be used to simulate networks with all-to-all connectivity and identical delays.

The simulation-time and firing-rate differences we observed between NEST and Brian2 suggest further investigations into both the performance and correctness of simulators (see also Van Albada et al., 2018). The all-to-all network due to Wang (2002) is likely not suitable as a reference benchmark for this purpose as all-to-all connectivity severely limits scalability and, for large networks, biological plausibility. Instead, the network model recently proposed by Moreni et al. (2025) might take on a similar role for models including NMDA-dynamics as the Potjans-Diesmann microcircuit model (Potjans & Diesmann, 2014) has had in driving simulation technology for networks composed of simpler neuron models (Senk et al., 2025).

The principal motivation behind developing an approximate model is to enable the study of networks of arbitrary topology. We applied the approximate model to a sparsely connected decision-making network, to see how sparseness affects the dynamics of the network. The fully connected decision making network has been studied extensively both numerically and analytically (Brunel & Wang, 2001; Wang, 2002; Wong & Wang, 2006). For models with instantaneous synapses, a complete mean-field theory has been developed (Amit & Brunel, 1997; Brunel, 2000). For the synaptic models studied here, a set of mean-field equations of the population firing rates of the fully connected decision making network can be constructed under a series of approximations, one of which is that the main sources of variation in the membrane potential are the AMPA synapses activated by the external Poisson population (Brunel & Wang, 2001). In a sparse decision-making network, there is significant variation also in the recurrent GABA and NMDA synapses, which have different time constants. In this setting, the same mathematical approach cannot be taken, and numerical simulations must be relied on instead.

We have shown that the ability of the sparse network to exhibit decision-making behavior is highly sensitive to the degree of connectivity, particularly the balance between the intra-selective connectivity and the inhibitory-selective connectivity (Fig. 6). For the particular parameter values chosen here, we found that there is a range of intra-selective and inhibitory-selective connectivities that support decision-making dynamics, and which influence the level of asymmetry in activity between the two selective populations. In particular, high intra-selective connectivities of over 50% were required, and significantly lower inhibitory-selective connectivities of around 25%–40%. The exact balance is also dependent on the synaptic strengths and conductances, which were not systematically varied in the same experiment.

## Conclusion

The approximate model of NMDA-receptor-mediated synaptic currents in leaky integrate-and-fire neurons presented in this paper shows behavior close to that of the original model by Wang and Brunel (Wang, 1999; Brunel & Wang, 2001; Wang, 2002). Synaptic currents in the exact and approximate model differ only for a few milliseconds after each incoming spike, leaving the dynamics of networks largely unaffected. While the original model is widely used, its practical applications are mostly restricted to fully connected networks with identical delays. By reducing simulation times by more than two orders of magnitude, our approximation to NMDA dynamics enables the investigation of more general networks of relevant size in close to real time without imposing restrictions on network connectivity or the distribution of synaptic delays.

## Data Availability

No datasets were generated or analysed during the current study.

## Footnotes

1 Firing rates in simulations performed with Brian2 are approximately 10% lower than those obtained with NEST for the exact model. We plan to investigate this elsewhere.

## Code Availability

The code used to generate the data and figures are available at: https://github.com/janskaar/approximate_NMDA_model
